# Crystal structures of [(*N*,*N*-di­methyl­amino)­meth­yl]ferrocene and (*R*_p_,*R*_p_)-bis­{2-[(di­methyl­amino)­meth­yl]ferrocen­yl}di­methyl­silane

**DOI:** 10.1107/S2056989020010397

**Published:** 2020-08-11

**Authors:** Anna Krupp, Jessica Wegge, Felix Otte, Johannes Kleinheider, Helene Wall, Carsten Strohmann

**Affiliations:** a Technische Universität Dortmund, Fakultät Chemie und Chemische Biologie, Otto-Hahn-Strasse 6, 44227 Dortmund, Germany

**Keywords:** crystal structure, ferrocene derivative, Hirshfeld surface analysis

## Abstract

The title compound [(*N*,*N*-di­methyl­amino)­meth­yl]ferrocene, [Fe(C_5_H_5_)(C_8_H_12_N)], (**1**), is an inter­esting starting material for the synthesis of planar chiral 1,2-disubstituted ferrocenes, as demonstrated by the preparation of (*R*_p_,*R*_p_)-bis­{2-[(di­methyl­amino)­meth­yl]ferrocen­yl}di­methyl­silane, [Fe_2_(C_5_H_5_)_2_(C_18_H_18_N_2_Si)], (**2**), from the li­thia­ted derivative of **1**. The configuration of the lithium compound is unchanged after the substitution reaction and the chirality is preserved in space group *P*2_1_2_1_2_1_. In both compounds, the Cp rings adopt eclipsed conformations. Hirshfeld surface analysis was used to investigate the inter­molecular inter­actions, and showed that H⋯H (van der Waals) inter­actions dominate in both structures with contact percentages of 83.9 and 88.4% for **1** and **2**, respectively.

## Abstract 2

The cyclo­penta­dienyl rings are eclipsed in both of the title compounds and their packing is dominated by H⋯H (van der Waals) contacts, as determined by Hirshfeld surface analyses.

## Chemical context   

In 1951, ferrocene was synthesized serendipitously (Kealy & Pauson, 1951 ▸) and one year later it was examined by X-ray crystallography (Fischer & Pfab, 1952 ▸). *N*,*N*-Di­methyl­amino­methyl­ferrocene (C_13_H_17_FeN,**1**) was first synthesized by Hauser & Lindsay (1956 ▸) by the reaction of ferrocene with paraformaldehyde and *N*,*N,N′*,*N′-*tetra­methyldi­amino­methane. The derivatization of ferrocene to planar chiral ferrocene makes it an important ligand for catalytic asymmetric transformations, both for scientific and industrial applications (Schaarschmidt & Lang, 2013 ▸). In particular, **1** is appropriate for the formation of 1,2-disubstituted ferrocenes because of the free electron pair at the nitro­gen atom: the li­thia­tion of the *ortho-*position is preferred due to the *DoM* effect (*Directed ortho Metalation*) and can be converted by a further step using an electrophile (Marr *et al.*, 1967 ▸). The *ortho*-li­thia­tion can be carried out both racemically or with a high degree of enanti­omeric control. The best known example for *ortho*-li­thia­tion with high stereoselectivity is the (*R*)-*N*,*N*-dimethyl-1-ferrocenyl­ethyl­amine, or Ugi’s amine with a chiral directing group (Marquarding *et al.*, 1970 ▸).

Many applications based on **1** have been established by our research group: it is an inexpensive non-chiral analogue of Ugi’s amine, therefore the desymmetrization must be implemented by the chiral auxiliary (*R*,*R*)-tetra­methyl-1,2-cyclo­hexa­nedi­amine (TMCDA) with yields in high stereoselectivity (Steffen *et al.*, 2013 ▸). One application of the 1,2-disubstituted ferrocenes based on **1** is the formation of racemic and enanti­omerically pure siloxides of zinc, whereby disiloxanes can be synthesized while avoiding condensation reactions (Golz *et al.*, 2017 ▸). Another application is the kinetically controlled asymmetric synthesis of silicon-stereogenic meth­oxy silanes using a planar chiral ferrocene backbone based on **1**. Here, silicon-stereogenic meth­oxy silanes could be prepared with excellent stereoinduction (*d.r*. > 99:1) and the mechanistic course of the reaction can be described by quantum-chemical calculations (Barth *et al.*, 2019 ▸). Nayyar *et al.* (2018 ▸) reported 1,2-disubstituted ferrocenes based on **1** and their use as precursors for the diastereoselective synthesis of divalent-element chlorides and an unprecedented organolithium-induced carbon–carbon single-bond cleavage. Furthermore, Gawron *et al.* (2019 ▸) were able to synthesize *N*,*N*-di­methyl­amino­methyl­ferrocene-backboned unsymmetrical pincer-type proligands, which are inter­esting as ligands for transition-metal complexes as catalysts for a variety of reactions in organic chemistry. The (*R*,*S*)-*meso*-compound of bis­[dimeth­yl(amino­meth­yl)ferrocen­yl]di­methyl­silane was characterized by Roewer and co-workers using X-ray diffraction analysis and formed during the synthesis of di­methyldi­chloro­silane with two equivalents of the racemic li­thia­ted *N*,*N*-di­methyl­amino­methyl­ferrocene (Palitzsch *et al.*, 1999 ▸).
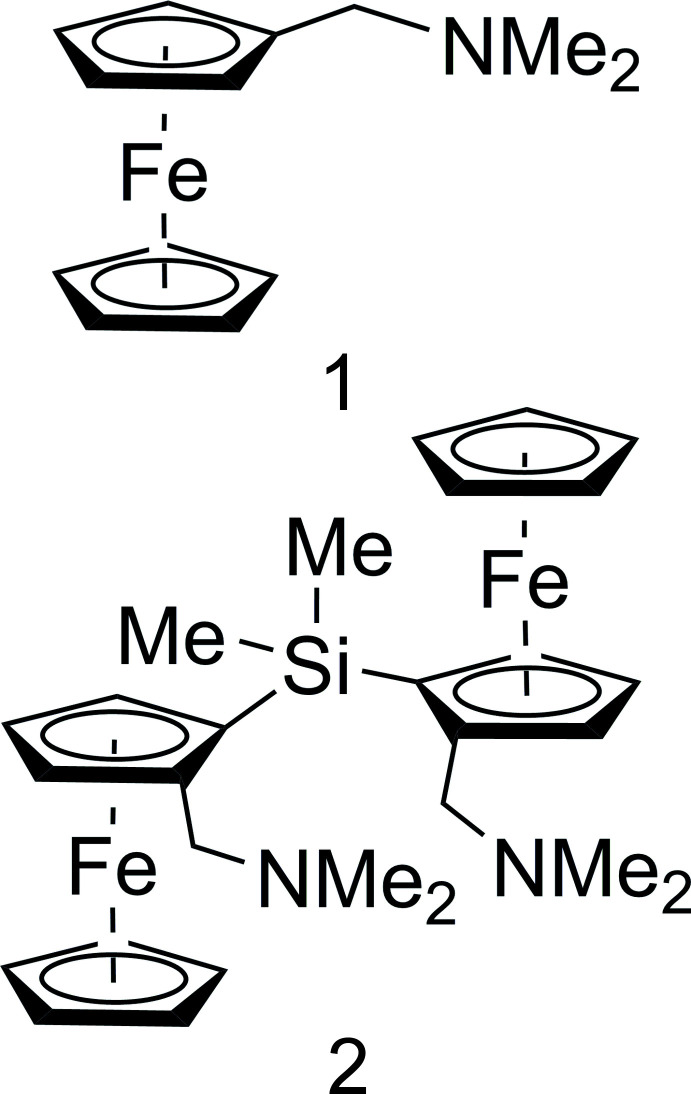


In this paper, we report the crystal structures of **1** and enantiomerically pure (*R*_p_,*R*_p_)-bis­[dimeth­yl(amino­meth­yl)ferro­cen­yl]di­methyl­silane (**2**) and analyze their inter­molecular inter­actions using Hirshfeld surfaces and two-dimensional fingerprint plots.

## Structural commentary   

**Figure 1:**
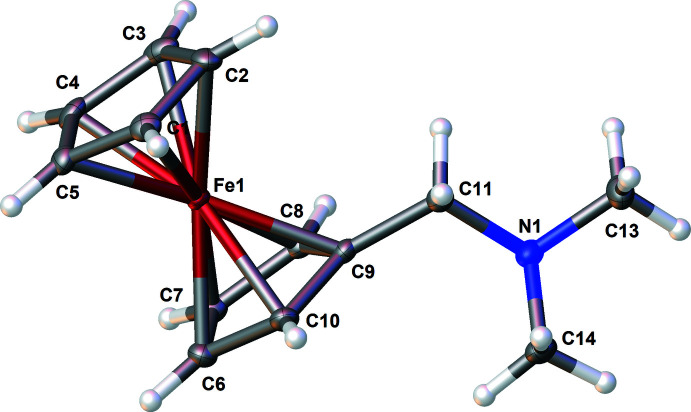
The mol­ecular structure of **1** showing 50% displacement ellipsoids.

Compound **1** crystallizes from *n*-pentane at 243 K as orange needles with monoclinic (*P*2_1_/*n*) symmetry. There are no noticeable irregularities in the bond lengths or bond angles found: the amino­methyl side chain is oriented above its attached cyclo­penta­dienyl ring, and the Cp rings are eclipsed, the dihedral angle between their mean planes being 1.53 (15)°. The mol­ecular structure of **1** is presented in Fig. 1 ▸.

**Figure 2:**
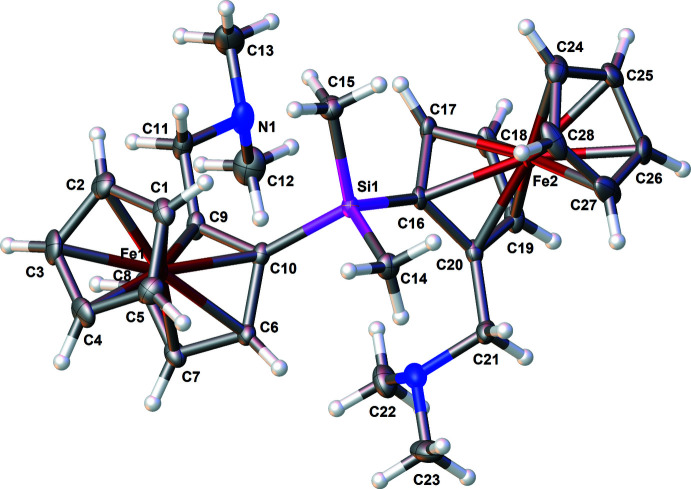
The mol­ecular structure of **2** showing 50% displacement ellipsoids.

Compound **2** is an orange–red crystalline solid and occurs in enantiomerically pure form in the ortho­rhom­bic space group *P*2_1_2_1_2_1_. The structure is illustrated in Fig. 2 ▸. Using Cahn–Ingold–Prelog (CIP) prioritization, compound **2** can be assigned the (*R*_p_,*R*_p_)-configuration; furthermore the cyclo­penta­dienyl rings are also in an eclipsed conformation for both iron atoms [dihedral angles = 4.89 (17) and 1.34 (18)° for the Fe1 and Fe2 rings, respectively]. The Si—C bonds span the range of 1.869 (3) to 1.874 (3) Å, which is consistent with the literature (Allen *et al.*, 1987 ▸). The silicon centre of compound **2** adopts a slightly distorted tetra­hedral geometry, as shown by the angles of 105.43 (14)° (C14—Si1—C10) as the smallest and 112.17 (13)° (C14—Si1—C16) as the largest. This flexibility is often observed for Si—C bonds (Otte *et al.*, 2017 ▸). Compared to compound **1**, the amino­methyl side chains are oriented in the direction of the silicon atom, but the N⋯Si contact distances of 3.552 (3) for N2 and 3.584 (3) Å for N1 are too long to be regarded as coordinate bonds to Si from the N lone pairs.

## Supra­molecular features   

**Figure 3:**
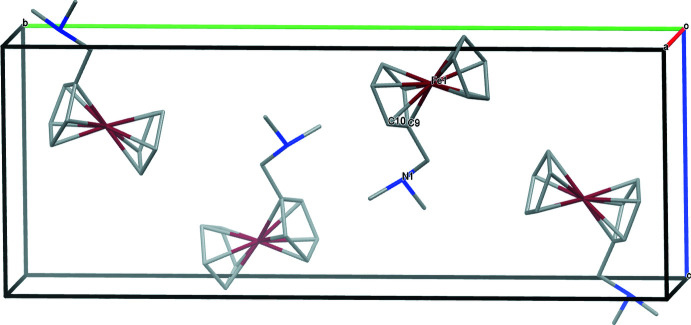
A view along the *a*-axis direction of the crystal packing of **1**.

**Figure 4:**
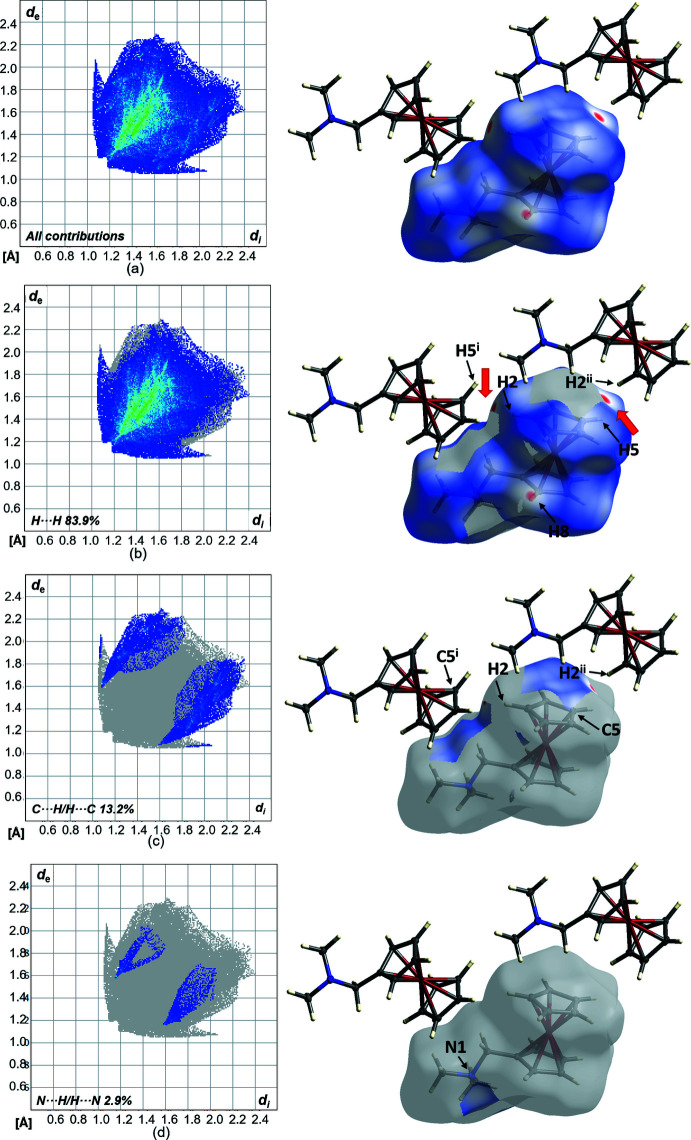
(*a*) Hirshfeld surfaces and two-dimensional fingerprint plots (*CrystalExplorer17*; Turner *et al.*, 2017 ▸; McKinnon *et al.*, 2007 ▸) of **1** showing close contacts in the crystal. (*b*)–(*d*) indicate the contributions of atoms within specific inter­acting pairs (blue areas). Symmetry codes: (i) *x* − 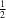, −*y* + 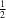, *z* + 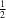; (ii) *x* + 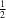, −*y* + 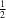, *z* − 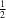.

The crystal packing of compound **1** is shown in Fig. 3 ▸. To further investigate close contacts and inter­molecular inter­actions, a Hirshfeld surface analysis was carried out: Fig. 4 ▸ illustrates the Hirshfeld surface mapped over *d*_norm_ in the range from −0.072 to 1.201 (arbitrary units) and the related fingerprint plots generated by *CrystalExplorer* (Turner *et al.*, 2017 ▸; McKinnon *et al.*, 2007 ▸). On the Hirshfeld surface, weak van der Waals H⋯H contacts appear as by far the largest region (83.9%) and show significant red spots on the Hirshfeld surface. C⋯H/H⋯C contacts contribute to 13.2% of the Hirshfeld area and appear as two spikes and also show a slight colouration, which indicates that the cyclo­penta­dienyl ring inter­acts with adjacent mol­ecules. The N⋯H/H⋯N inter­actions occupy the smallest region (2.9%) and display no noticeable inter­actions.

**Figure 5:**
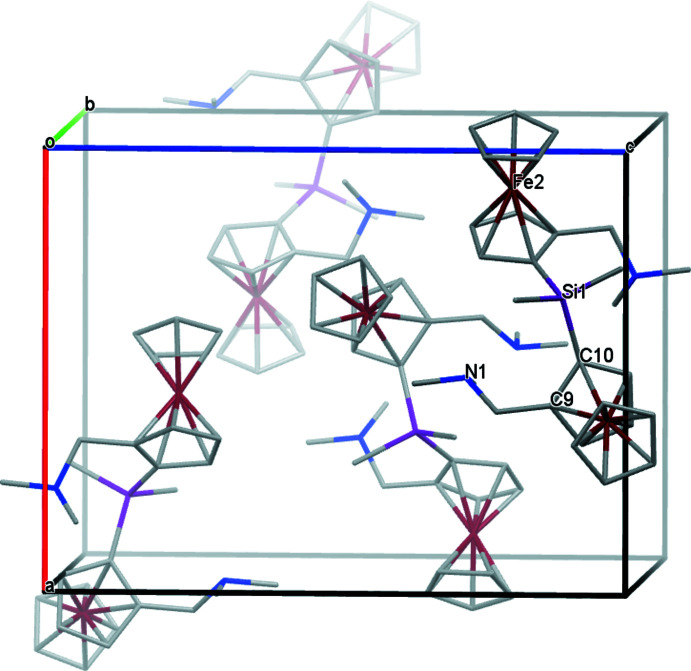
A view along the *b-*axis direction of the crystal packing of **2**.

**Figure 6:**
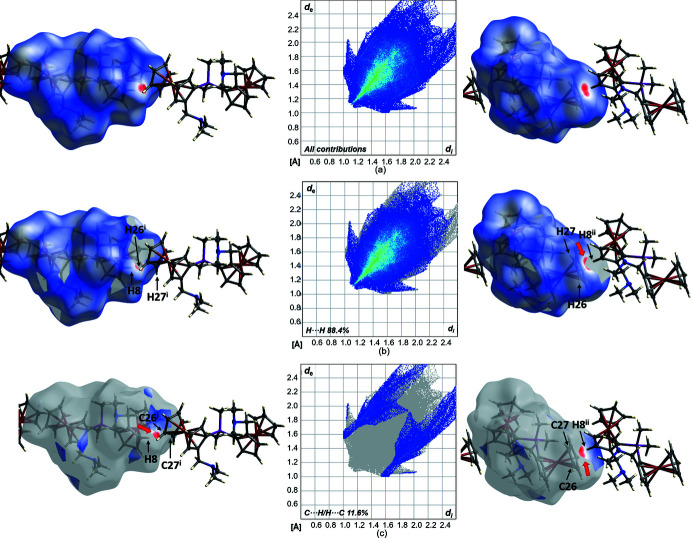
(*a*) Hirshfeld surfaces and two-dimensional fingerprint plots (*CrystalExplorer17*; Turner *et al.*, 2017 ▸; McKinnon *et al.*, 2007 ▸) of compound **2** showing close contacts in the crystal. (*b*) and (*c*) indicate the contributions of atoms within specific inter­acting pairs (blue areas). Symmetry codes: (i) *x* − 1, *y*, *z*; (ii) *x* + 1, *y*, *z*.

The crystal packing of compound **2** is illustrated in Fig. 5 ▸. The Hirshfeld surfaces and contributions of the different types of inter­molecular inter­actions are shown in Fig. 6 ▸ in the two-dimensional fingerprint plot. The Hirshfeld surface of compound **2** mapped over *d*_norm_ in the range from −0.149 to 1.497 a.u. shows significant inter­molecular inter­actions, indicated by the red spots. Both the van der Waals H⋯H contacts (88.4%) and the C⋯H/H⋯C contacts (11.6%) contribute to the packing arrangement of the crystal. Inter­molecular inter­actions of the cyclo­penta­dienyl rings with neighbouring mol­ecules can also be visualized.

## Database survey   

There are a large number of compounds based on **1**. Selected examples found in the Cambridge Structural Database (CSD, version 5.41, update of May 2020; Groom *et al.*, 2016 ▸) include (*R*,*S*)-meso-bis­[dimeth­yl(amino­meth­yl)ferrocen­yl]di­methyl­silane (CSD refcode KENRUQ; Palitzsch *et al.*, 1999 ▸), (*R*,*S*)-*meso*-bis­[dimeth­yl(amino­meth­yl)ferrocen­yl]di­chloro­silane (KENQUP; Palitzsch *et al.*, 1999 ▸), the monoetherate of the homochiral dimer (*S*_p_)-[2-(di­methyl­amino­meth­yl)ferrocen­yl]lithium (LISBOG; Steffen *et al.*, 2013 ▸), bis­[μ-{2-[(di­methyl­ammonium­yl)meth­yl]ferrocen­yl}(dimeth­yl)silanolato]tetra­chloro­dizinc(II) (FAWPIF; Golz *et al.*, 2017 ▸), [dimeth­yl(amino­meth­yl)ferrocen­yl]meth­oxy­methyl­phenyl­silane (SOKDAA; Barth *et al.*, 2019 ▸), 2-(di­methyl­amino­meth­yl)-1-{1-[(2,6-di-iso­propyl­phen­yl)amino]-2,2-di­methyl­prop­yl}-3-(tri­methyl­sil­yl)ferrocene (RIGDOD; Nayyar *et al.*, 2018 ▸) and 1-bromo-2-(di­phenyl­phosphino)-5-[(di­methyl­amino)­meth­yl]ferrocene (MIZMOA; Gawron *et al.*, 2019 ▸).

## Synthesis and crystallization   

*N*,*N*-Di­methyl­amino­methyl­ferrocene was purchased from ABCR and used without further purification. A solution of *N*,*N*-di­methyl­amino­methyl­ferrocene (1.00 mmol) in *n*-pentane (1 ml) was made up and stored at 243 K and compound **1** crystallized in the form of orange needles.

**Figure 7:**
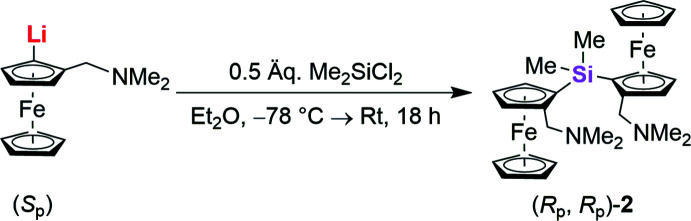
Reaction scheme for the synthesis of **2**.

The reaction scheme for the synthesis of compound **2** is illustrated in Fig. 7 ▸. To a solution of (*S*_p_)-[2-(di­methyl­amino­meth­yl)ferrocen­yl]lithium (4.00 mmol) (Steffen *et al.*, 2013 ▸) in diethyl ether, di­methyldi­chloro­silane (2.00 mmol) was added dropwise at 195 K. The reaction was slowly warmed up to room temperature and stirred overnight. Afterwards the reaction was quenched by the addition of water. The aqueous phase was extracted three times with diethyl ether and the combined organic phases were dried with MgSO_4_. After the volatile components were removed and purified by column chromatography (*n*-penta­ne:diethyl ether + tri­ethyl­amine; 100:1 + 5 Vol.-%), the product (46%) could be obtained as yellowish plates.

^1^H NMR (600.3 MHz, C_6_D_6_): *δ* = 0.81 [*s*, 6H; Si(C*H*_3_)_2_], 2.02 {*s*, 12H; [N(C*H*_3_)_2_]_2_}, 2.80, 3.64 [AB-system, 4H, ^2^ *J*_HH_ = 12.3 Hz; CpC*H*_2_N)_2_], 4.08 [*s*, 10H; (Cp—C*H_2_*)], 4.12 [*m*, 2H; (Cp—C*H_2_*)], 4.19 [*m*, 2H; (Cp—C*H*)_2_], 4.35 [*m*, 2H; Cp—C*H*)_2_] ppm.

{^1^H}^13^C NMR (150.9 MHz, C_6_D_6_): *δ* = 0.6 [2C; Si(*C*H_3_)_2_], 45.4 {4C; [CH_2_N(*C*H_3_)_2_]_2_}, 60.5 [2C; (Cp*C*H_2_N)_2_], 69.7 [10C; (Cp—*C*H)_2_], 69.8 [2C; (Cp—*C*H)_2_], 72.6 [2C; (Cp—*C*)_2_Si)], 74.2 [2C; (Cp—*C*H)_2_], 76.6 [2C; (Cp—*C*H)_2_], 90.5 [2C; (Cp—*C*CH_2_N)_2_] ppm.

{^1^H}^29^Si NMR (119.3 MHz, C_6_D_6_): *δ* = −7.07 [*s*, 1Si; *Si*(CH_3_)_2_] ppm.

ESI-(+)-MS: *m*/*z* (%): 498 (20) [(*M*–NMe_2_)^+^], 409 (100) [(*M*–NMe_2_–CH_2_NMe_2_–Me_2_)^+^], 299 (50) [(*M*–FcCH_2_NMe_2_)^+^], 199 (50) [(*M*–SiMe_2_FcCH_2_NMe_2_–NMe_2_)^+^].

*R*_f:_ (*n*-penta­ne: Et_2_O + Et_3_N; 100: 1 + 5 Vol.-%) = 0.20.

## Refinement   

Crystal data, data collection and structure refinement details are summarized in Table 1 ▸. For both compounds, the H atoms were positioned geometrically (C—H = 0.95–1.00 Å) and refined using a riding model, with *U*_iso_(H) = 1.2*U*_eq_(C) for CH_2_ and CH hydrogen atoms and *U*_iso_(H) = 1.5*U*_eq_(C) for CH_3_ hydrogen atoms.

## Supplementary Material

Crystal structure: contains datablock(s) 1, 2, global. DOI: 10.1107/S2056989020010397/hb7933sup1.cif

Structure factors: contains datablock(s) 1. DOI: 10.1107/S2056989020010397/hb79331sup2.hkl

Structure factors: contains datablock(s) 2. DOI: 10.1107/S2056989020010397/hb79332sup3.hkl

CCDC references: 2019451, 2019450

Additional supporting information:  crystallographic information; 3D view; checkCIF report

## Figures and Tables

**Table 1:** Experimental details

	**1**	**2**
Crystal data		
Chemical formula	[Fe(C_5_H_5_)(C_8_H_12_N)]	[Fe_2_(C_5_H_5_)_2_(C_18_H_18_N_2_Si)]
*M*_r_	243.12	542.39
Crystal system, space group	Monoclinic, *P*2_1_/*n*	Orthorhombic, *P*2_1_2_1_2_1_
Temperature (K)	100	100
*a*, *b*, *c* (Å)	5.6777 (3), 23.0873 (15), 8.7206 (6)	12.0132 (7), 14.0683 (8), 15.7169 (11)
α, β, γ (°)	90, 90.590 (2), 90	90, 90, 90
*V* (Å^3^)	1143.06 (12)	2656.2 (3)
*Z*	4	4
Radiation type	Mo *K*α	Cu *K*α
μ (mm^−1^)	1.28	9.32
Crystal size (mm)	0.55 × 0.22 × 0.19	0.47 × 0.23 × 0.08

Data collection		
Diffractometer	Bruker D8 Venture	Bruker D8 Venture
Absorption correction	Multi-scan (*SADABS*; Bruker, 2016 ▸)	Multi-scan (*SADABS*; Bruker, 2016 ▸)
*T*_min_, *T*_max_	0.351, 0.435	0.326, 0.754
No. of measured, independent and observed [*I* > 2σ(*I*)] reflections	19466, 3960, 3489	41156, 5733, 5518
*R*_int_	0.040	0.060
(sin θ/λ)_max_ (Å^−1^)	0.746	0.639

Refinement		
*R*[*F* ^2^ > 2σ(*F* ^2^)], *wR*(*F* ^2^), *S*	0.047, 0.118, 1.17	0.028, 0.065, 1.07
No. of reflections	3960	5733
No. of parameters	138	304
H-atom treatment	H-atom parameters constrained	H-atom parameters constrained
Δρ_max_, Δρ_min_ (e Å^−3^)	1.34, −1.47	0.40, −0.27
Absolute structure	–	Flack *x* determined using 2287 quotients [(*I* ^+^)−(*I* ^−^)]/[(*I* ^+^)+(*I* ^−^)] (Parsons *et al.*, 2013 ▸)
Absolute structure parameter	–	−0.009 (2)

Computer programs: *APEX2* (Bruker, 2018 ▸), *SAINT* (Bruker, 2016 ▸), *SHELXT* (Sheldrick, 2015*b* ▸), *SHELXS* (Sheldrick, 2008 ▸), *SHELXL* (Sheldrick, 2015*a* ▸), *OLEX2* (Dolomanov *et al.*, 2009 ▸), *publCIF* (Westrip, 2010 ▸) and *Mercury* (Macrae *et al.*, 2020 ▸).

## supplementary crystallographic information

### [(N,N-Dimethylamino)methyl]ferrocene (1) Crystal data

**Table:**

[Fe(C_5_H_5_)(C_8_H_12_N)]	*F*(000) = 512
*M**_r_* = 243.12	*D*_x_ = 1.413 Mg m^−^^3^
Monoclinic, *P*2_1_/*n*	Mo *K*α radiation, λ = 0.71073 Å
*a* = 5.6777 (3) Å	Cell parameters from 9923 reflections
*b* = 23.0873 (15) Å	θ = 2.5–33.1°
*c* = 8.7206 (6) Å	µ = 1.28 mm^−^^1^
β = 90.590 (2)°	*T* = 100 K
*V* = 1143.06 (12) Å^3^	Needle, clear orange
*Z* = 4	0.55 × 0.22 × 0.19 mm

### [(N,N-Dimethylamino)methyl]ferrocene (1) Data collection

**Table:**

Bruker D8 Venture diffractometer	3960 independent reflections
Radiation source: Advanced Light Source, station 11.3.1, Incoatec Iµs	3489 reflections with *I* > 2σ(*I*)
Silicon 111 monochromator	*R*_int_ = 0.040
Detector resolution: 10.42 pixels mm^-1^	θ_max_ = 32.0°, θ_min_ = 2.5°
φ and ω scans	*h* = −8→8
Absorption correction: multi-scan (SADABS; Bruker, 2016)	*k* = −34→33
*T*_min_ = 0.351, *T*_max_ = 0.435	*l* = −13→13
19466 measured reflections	

### [(N,N-Dimethylamino)methyl]ferrocene (1) Refinement

**Table:**

Refinement on *F*^2^	Primary atom site location: dual
Least-squares matrix: full	Hydrogen site location: inferred from neighbouring sites
*R*[*F*^2^ > 2σ(*F*^2^)] = 0.047	H-atom parameters constrained
*wR*(*F*^2^) = 0.118	*w* = 1/[σ^2^(*F*_o_^2^) + (0.0175*P*)^2^ + 3.606*P*] where *P* = (*F*_o_^2^ + 2*F*_c_^2^)/3
*S* = 1.17	(Δ/σ)_max_ = 0.001
3960 reflections	Δρ_max_ = 1.34 e Å^−^^3^
138 parameters	Δρ_min_ = −1.47 e Å^−^^3^
0 restraints	

### [(N,N-Dimethylamino)methyl]ferrocene (1) Special details

**Table:**

Geometry. All esds (except the esd in the dihedral angle between two l.s. planes) are estimated using the full covariance matrix. The cell esds are taken into account individually in the estimation of esds in distances, angles and torsion angles; correlations between esds in cell parameters are only used when they are defined by crystal symmetry. An approximate (isotropic) treatment of cell esds is used for estimating esds involving l.s. planes.

### [(N,N-Dimethylamino)methyl]ferrocene (1) Fractional atomic coordinates and isotropic or equivalent isotropic displacement parameters (Å^2^)

**Table:**

	*x*	*y*	*z*	*U*_iso_*/*U*_eq_	
Fe1	0.86749 (6)	0.35631 (2)	0.15193 (4)	0.01111 (9)	
N1	0.4929 (4)	0.41321 (9)	0.5681 (2)	0.0171 (4)	
C1	1.1153 (4)	0.29284 (11)	0.1819 (3)	0.0161 (4)	
H1	1.242570	0.293233	0.253451	0.019*	
C2	0.8841 (5)	0.27051 (10)	0.2089 (3)	0.0184 (5)	
H2	0.830272	0.253686	0.301558	0.022*	
C3	0.7492 (5)	0.27812 (11)	0.0714 (3)	0.0207 (5)	
H3	0.589384	0.267101	0.056315	0.025*	
C4	0.8951 (5)	0.30501 (11)	−0.0390 (3)	0.0187 (5)	
H4	0.849873	0.315056	−0.140783	0.022*	
C5	1.1209 (4)	0.31432 (11)	0.0292 (3)	0.0160 (4)	
H5	1.252361	0.331809	−0.018966	0.019*	
C6	0.9505 (5)	0.44290 (10)	0.1543 (3)	0.0162 (4)	
H6	1.081500	0.460538	0.106051	0.019*	
C7	0.7223 (5)	0.43439 (11)	0.0875 (3)	0.0168 (4)	
H7	0.674308	0.445562	−0.012877	0.020*	
C8	0.5801 (4)	0.40629 (10)	0.1978 (3)	0.0151 (4)	
H8	0.420223	0.395284	0.183084	0.018*	
C9	0.7161 (4)	0.39721 (10)	0.3345 (3)	0.0138 (4)	
C10	0.9467 (4)	0.42004 (10)	0.3076 (3)	0.0153 (4)	
H10	1.074704	0.420067	0.378663	0.018*	
C11	0.6260 (5)	0.37075 (10)	0.4794 (3)	0.0156 (4)	
H11A	0.523300	0.337387	0.453899	0.019*	
H11B	0.760267	0.356324	0.541817	0.019*	
C12	0.6491 (5)	0.45778 (11)	0.6315 (3)	0.0218 (5)	
H12A	0.558986	0.483504	0.698386	0.033*	
H12B	0.716922	0.480354	0.547672	0.033*	
H12C	0.775825	0.439342	0.690955	0.033*	
C13	0.3659 (5)	0.38483 (12)	0.6917 (3)	0.0221 (5)	
H13A	0.258109	0.355844	0.648437	0.033*	
H13B	0.275809	0.413759	0.748726	0.033*	
H13C	0.478642	0.365806	0.761125	0.033*	

### [(N,N-Dimethylamino)methyl]ferrocene (1) Atomic displacement parameters (Å^2^)

**Table:**

	*U*^11^	*U*^22^	*U*^33^	*U*^12^	*U*^13^	*U*^23^
Fe1	0.01275 (15)	0.00967 (14)	0.01093 (14)	0.00032 (12)	0.00076 (10)	−0.00089 (11)
N1	0.0225 (10)	0.0131 (9)	0.0157 (9)	0.0018 (8)	0.0041 (8)	−0.0007 (7)
C1	0.0169 (10)	0.0137 (10)	0.0176 (11)	0.0032 (8)	−0.0003 (8)	−0.0004 (8)
C2	0.0235 (12)	0.0109 (10)	0.0209 (11)	−0.0015 (9)	0.0048 (9)	0.0013 (8)
C3	0.0164 (11)	0.0162 (11)	0.0297 (14)	0.0000 (9)	0.0004 (10)	−0.0078 (10)
C4	0.0235 (12)	0.0169 (11)	0.0157 (11)	0.0040 (9)	−0.0012 (9)	−0.0065 (8)
C5	0.0175 (10)	0.0150 (10)	0.0154 (10)	0.0024 (8)	0.0043 (8)	−0.0017 (8)
C6	0.0190 (11)	0.0124 (10)	0.0172 (11)	−0.0021 (8)	0.0022 (8)	0.0006 (8)
C7	0.0210 (11)	0.0134 (10)	0.0160 (10)	0.0026 (8)	0.0004 (8)	0.0011 (8)
C8	0.0159 (10)	0.0126 (10)	0.0167 (10)	0.0021 (8)	0.0003 (8)	−0.0033 (8)
C9	0.0157 (10)	0.0123 (9)	0.0135 (10)	0.0001 (8)	0.0009 (8)	−0.0019 (8)
C10	0.0162 (10)	0.0129 (10)	0.0166 (10)	−0.0027 (8)	0.0001 (8)	−0.0023 (8)
C11	0.0205 (11)	0.0117 (9)	0.0147 (10)	0.0009 (8)	0.0033 (8)	−0.0010 (8)
C12	0.0328 (14)	0.0144 (11)	0.0181 (11)	−0.0022 (10)	0.0022 (10)	−0.0037 (9)
C13	0.0260 (13)	0.0214 (12)	0.0190 (12)	0.0025 (10)	0.0078 (10)	0.0027 (9)

### [(N,N-Dimethylamino)methyl]ferrocene (1) Geometric parameters (Å, º)

**Table:**

Fe1—C1	2.046 (2)	C4—C5	1.424 (4)
Fe1—C2	2.044 (2)	C5—H5	0.9500
Fe1—C3	2.048 (3)	C6—H6	0.9500
Fe1—C4	2.051 (2)	C6—C7	1.429 (4)
Fe1—C5	2.047 (2)	C6—C10	1.437 (3)
Fe1—C6	2.054 (2)	C7—H7	0.9500
Fe1—C7	2.058 (2)	C7—C8	1.419 (3)
Fe1—C8	2.041 (2)	C8—H8	0.9500
Fe1—C9	2.048 (2)	C8—C9	1.429 (3)
Fe1—C10	2.049 (2)	C9—C10	1.433 (3)
N1—C11	1.464 (3)	C9—C11	1.498 (3)
N1—C12	1.463 (3)	C10—H10	0.9500
N1—C13	1.459 (3)	C11—H11A	0.9900
C1—H1	0.9500	C11—H11B	0.9900
C1—C2	1.432 (4)	C12—H12A	0.9800
C1—C5	1.422 (3)	C12—H12B	0.9800
C2—H2	0.9500	C12—H12C	0.9800
C2—C3	1.427 (4)	C13—H13A	0.9800
C3—H3	0.9500	C13—H13B	0.9800
C3—C4	1.419 (4)	C13—H13C	0.9800
C4—H4	0.9500		
			
C1—Fe1—C3	68.61 (11)	C4—C3—Fe1	69.85 (15)
C1—Fe1—C4	68.41 (10)	C4—C3—C2	108.2 (2)
C1—Fe1—C5	40.65 (10)	C4—C3—H3	125.9
C1—Fe1—C6	122.56 (10)	Fe1—C4—H4	126.5
C1—Fe1—C7	159.23 (10)	C3—C4—Fe1	69.63 (15)
C1—Fe1—C9	121.64 (10)	C3—C4—H4	125.9
C1—Fe1—C10	106.48 (10)	C3—C4—C5	108.2 (2)
C2—Fe1—C1	40.99 (10)	C5—C4—Fe1	69.50 (14)
C2—Fe1—C3	40.83 (11)	C5—C4—H4	125.9
C2—Fe1—C4	68.53 (11)	Fe1—C5—H5	126.1
C2—Fe1—C5	68.73 (10)	C1—C5—Fe1	69.66 (14)
C2—Fe1—C6	158.54 (11)	C1—C5—C4	108.0 (2)
C2—Fe1—C7	158.83 (11)	C1—C5—H5	126.0
C2—Fe1—C9	106.06 (10)	C4—C5—Fe1	69.81 (14)
C2—Fe1—C10	121.71 (10)	C4—C5—H5	126.0
C3—Fe1—C4	40.52 (11)	Fe1—C6—H6	126.4
C3—Fe1—C6	159.37 (11)	C7—C6—Fe1	69.81 (14)
C3—Fe1—C7	123.33 (11)	C7—C6—H6	126.1
C3—Fe1—C10	158.34 (11)	C7—C6—C10	107.8 (2)
C4—Fe1—C6	123.44 (11)	C10—C6—Fe1	69.30 (13)
C4—Fe1—C7	108.54 (10)	C10—C6—H6	126.1
C5—Fe1—C3	68.47 (10)	Fe1—C7—H7	126.8
C5—Fe1—C4	40.69 (10)	C6—C7—Fe1	69.52 (14)
C5—Fe1—C6	107.69 (10)	C6—C7—H7	126.1
C5—Fe1—C7	123.64 (10)	C8—C7—Fe1	69.13 (14)
C5—Fe1—C9	158.15 (10)	C8—C7—C6	107.9 (2)
C5—Fe1—C10	122.41 (10)	C8—C7—H7	126.1
C6—Fe1—C7	40.67 (10)	Fe1—C8—H8	125.9
C8—Fe1—C1	158.39 (10)	C7—C8—Fe1	70.38 (14)
C8—Fe1—C2	122.35 (10)	C7—C8—H8	125.5
C8—Fe1—C3	107.78 (10)	C7—C8—C9	109.0 (2)
C8—Fe1—C4	123.60 (10)	C9—C8—Fe1	69.78 (14)
C8—Fe1—C5	159.71 (10)	C9—C8—H8	125.5
C8—Fe1—C6	68.39 (10)	C8—C9—Fe1	69.30 (13)
C8—Fe1—C7	40.50 (10)	C8—C9—C10	107.2 (2)
C8—Fe1—C9	40.91 (9)	C8—C9—C11	125.3 (2)
C8—Fe1—C10	68.58 (10)	C10—C9—Fe1	69.57 (13)
C9—Fe1—C3	122.30 (10)	C10—C9—C11	127.5 (2)
C9—Fe1—C4	159.03 (10)	C11—C9—Fe1	128.12 (17)
C9—Fe1—C6	68.98 (10)	Fe1—C10—H10	126.4
C9—Fe1—C7	68.78 (10)	C6—C10—Fe1	69.69 (14)
C9—Fe1—C10	40.95 (9)	C6—C10—H10	126.0
C10—Fe1—C4	159.18 (11)	C9—C10—Fe1	69.48 (13)
C10—Fe1—C6	41.01 (10)	C9—C10—C6	108.0 (2)
C10—Fe1—C7	68.67 (10)	C9—C10—H10	126.0
C12—N1—C11	110.9 (2)	N1—C11—C9	110.8 (2)
C13—N1—C11	110.6 (2)	N1—C11—H11A	109.5
C13—N1—C12	109.8 (2)	N1—C11—H11B	109.5
Fe1—C1—H1	126.5	C9—C11—H11A	109.5
C2—C1—Fe1	69.42 (14)	C9—C11—H11B	109.5
C2—C1—H1	126.0	H11A—C11—H11B	108.1
C5—C1—Fe1	69.69 (14)	N1—C12—H12A	109.5
C5—C1—H1	126.0	N1—C12—H12B	109.5
C5—C1—C2	108.0 (2)	N1—C12—H12C	109.5
Fe1—C2—H2	126.1	H12A—C12—H12B	109.5
C1—C2—Fe1	69.59 (14)	H12A—C12—H12C	109.5
C1—C2—H2	126.2	H12B—C12—H12C	109.5
C3—C2—Fe1	69.73 (15)	N1—C13—H13A	109.5
C3—C2—C1	107.6 (2)	N1—C13—H13B	109.5
C3—C2—H2	126.2	N1—C13—H13C	109.5
Fe1—C3—H3	126.4	H13A—C13—H13B	109.5
C2—C3—Fe1	69.44 (14)	H13A—C13—H13C	109.5
C2—C3—H3	125.9	H13B—C13—H13C	109.5
			
Fe1—C1—C2—C3	59.61 (17)	C5—C1—C2—Fe1	−59.20 (17)
Fe1—C1—C5—C4	−59.49 (17)	C5—C1—C2—C3	0.4 (3)
Fe1—C2—C3—C4	59.32 (18)	C6—C7—C8—Fe1	−58.93 (17)
Fe1—C3—C4—C5	58.99 (17)	C6—C7—C8—C9	0.3 (3)
Fe1—C4—C5—C1	59.40 (17)	C7—C6—C10—Fe1	59.39 (17)
Fe1—C6—C7—C8	58.68 (17)	C7—C6—C10—C9	0.3 (3)
Fe1—C6—C10—C9	−59.09 (16)	C7—C8—C9—Fe1	−59.63 (17)
Fe1—C7—C8—C9	59.27 (17)	C7—C8—C9—C10	−0.2 (3)
Fe1—C8—C9—C10	59.47 (16)	C7—C8—C9—C11	177.6 (2)
Fe1—C8—C9—C11	−122.8 (2)	C8—C9—C10—Fe1	−59.31 (16)
Fe1—C9—C10—C6	59.22 (17)	C8—C9—C10—C6	−0.1 (3)
Fe1—C9—C11—N1	−169.70 (17)	C8—C9—C11—N1	−79.4 (3)
C1—C2—C3—Fe1	−59.52 (17)	C10—C6—C7—Fe1	−59.07 (17)
C1—C2—C3—C4	−0.2 (3)	C10—C6—C7—C8	−0.4 (3)
C2—C1—C5—Fe1	59.04 (17)	C10—C9—C11—N1	97.9 (3)
C2—C1—C5—C4	−0.5 (3)	C11—C9—C10—Fe1	123.0 (2)
C2—C3—C4—Fe1	−59.06 (18)	C11—C9—C10—C6	−177.8 (2)
C2—C3—C4—C5	−0.1 (3)	C12—N1—C11—C9	−69.5 (3)
C3—C4—C5—Fe1	−59.07 (18)	C13—N1—C11—C9	168.4 (2)
C3—C4—C5—C1	0.3 (3)		

### (Rp,Rp)-Bis{2-[(dimethylamino)methyl]ferrocenyl}dimethylsilane (2) Crystal data

**Table:**

[Fe_2_(C_5_H_5_)_2_(C_18_H_18_N_2_Si)]	*D*_x_ = 1.356 Mg m^−^^3^
*M**_r_* = 542.39	Cu *K*α radiation, λ = 1.54178 Å
Orthorhombic, *P*2_1_2_1_2_1_	Cell parameters from 9834 reflections
*a* = 12.0132 (7) Å	θ = 4.2–78.6°
*b* = 14.0683 (8) Å	µ = 9.32 mm^−^^1^
*c* = 15.7169 (11) Å	*T* = 100 K
*V* = 2656.2 (3) Å^3^	Plate, clear orangish yellow
*Z* = 4	0.47 × 0.23 × 0.08 mm
*F*(000) = 1144	

### (Rp,Rp)-Bis{2-[(dimethylamino)methyl]ferrocenyl}dimethylsilane (2) Data collection

**Table:**

Bruker D8 Venture diffractometer	5733 independent reflections
Radiation source: microfocus sealed X-ray tube, Incoatec Iµs	5518 reflections with *I* > 2σ(*I*)
HELIOS mirror optics monochromator	*R*_int_ = 0.060
Detector resolution: 10.4167 pixels mm^-1^	θ_max_ = 79.9°, θ_min_ = 4.2°
ω and φ scans	*h* = −13→15
Absorption correction: multi-scan (SADABS; Bruker, 2016)	*k* = −17→17
*T*_min_ = 0.326, *T*_max_ = 0.754	*l* = −20→17
41156 measured reflections	

### (Rp,Rp)-Bis{2-[(dimethylamino)methyl]ferrocenyl}dimethylsilane (2) Refinement

**Table:**

Refinement on *F*^2^	Hydrogen site location: inferred from neighbouring sites
Least-squares matrix: full	H-atom parameters constrained
*R*[*F*^2^ > 2σ(*F*^2^)] = 0.028	*w* = 1/[σ^2^(*F*_o_^2^) + (0.0111*P*)^2^ + 2.2762*P*] where *P* = (*F*_o_^2^ + 2*F*_c_^2^)/3
*wR*(*F*^2^) = 0.065	(Δ/σ)_max_ = 0.001
*S* = 1.07	Δρ_max_ = 0.40 e Å^−^^3^
5733 reflections	Δρ_min_ = −0.27 e Å^−^^3^
304 parameters	Absolute structure: Flack *x* determined using 2287 quotients [(*I*^+^)-(*I*^-^)]/[(*I*^+^)+(*I*^-^)] (Parsons *et al.*, 2013)
0 restraints	Absolute structure parameter: −0.009 (2)
Primary atom site location: structure-invariant direct methods	

### (Rp,Rp)-Bis{2-[(dimethylamino)methyl]ferrocenyl}dimethylsilane (2) Special details

**Table:**

Geometry. All esds (except the esd in the dihedral angle between two l.s. planes) are estimated using the full covariance matrix. The cell esds are taken into account individually in the estimation of esds in distances, angles and torsion angles; correlations between esds in cell parameters are only used when they are defined by crystal symmetry. An approximate (isotropic) treatment of cell esds is used for estimating esds involving l.s. planes.

### (Rp,Rp)-Bis{2-[(dimethylamino)methyl]ferrocenyl}dimethylsilane (2) Fractional atomic coordinates and isotropic or equivalent isotropic displacement parameters (Å^2^)

**Table:**

	*x*	*y*	*z*	*U*_iso_*/*U*_eq_	
Fe1	0.40210 (4)	0.41292 (3)	0.52611 (3)	0.01371 (10)	
Fe2	0.91114 (4)	0.56363 (3)	0.70011 (3)	0.01381 (10)	
Si1	0.67323 (6)	0.46262 (5)	0.60692 (5)	0.01300 (16)	
N1	0.4872 (2)	0.5234 (2)	0.77668 (18)	0.0250 (6)	
N2	0.7054 (2)	0.67859 (19)	0.49626 (19)	0.0226 (6)	
C1	0.4524 (3)	0.2732 (2)	0.5230 (2)	0.0206 (6)	
H1	0.522465	0.249185	0.541009	0.025*	
C2	0.3558 (3)	0.2845 (2)	0.5749 (2)	0.0230 (7)	
H2	0.350364	0.269161	0.633668	0.028*	
C3	0.2693 (3)	0.3224 (2)	0.5237 (3)	0.0261 (7)	
H3	0.195895	0.336837	0.542013	0.031*	
C4	0.3122 (3)	0.3350 (2)	0.4401 (2)	0.0250 (7)	
H4	0.272369	0.359429	0.392671	0.030*	
C5	0.4250 (3)	0.3045 (2)	0.4397 (2)	0.0221 (7)	
H5	0.473605	0.305010	0.392052	0.027*	
C6	0.4964 (2)	0.5297 (2)	0.4952 (2)	0.0160 (6)	
H6	0.545571	0.535356	0.448062	0.019*	
C7	0.3812 (2)	0.5528 (2)	0.4941 (2)	0.0200 (6)	
H7	0.340403	0.576119	0.446778	0.024*	
C8	0.3381 (3)	0.5346 (2)	0.5769 (2)	0.0209 (7)	
H8	0.263383	0.544423	0.594646	0.025*	
C9	0.4271 (2)	0.4989 (2)	0.6289 (2)	0.0171 (6)	
C10	0.5271 (2)	0.4965 (2)	0.5782 (2)	0.0151 (6)	
C11	0.4148 (3)	0.4689 (2)	0.7201 (2)	0.0218 (6)	
H11A	0.336404	0.477614	0.737880	0.026*	
H11B	0.432944	0.400471	0.725163	0.026*	
C12	0.4651 (4)	0.6248 (3)	0.7704 (3)	0.0358 (9)	
H12A	0.476258	0.645725	0.711601	0.054*	
H12B	0.515962	0.659448	0.808016	0.054*	
H12C	0.388032	0.637477	0.787533	0.054*	
C13	0.4753 (3)	0.4906 (3)	0.8634 (2)	0.0353 (9)	
H13A	0.398706	0.501402	0.882532	0.053*	
H13B	0.526785	0.525684	0.900183	0.053*	
H13C	0.492332	0.422561	0.866214	0.053*	
C14	0.7422 (2)	0.4315 (2)	0.50393 (19)	0.0188 (6)	
H14A	0.699785	0.381123	0.475523	0.028*	
H14B	0.818102	0.409171	0.515008	0.028*	
H14C	0.744808	0.487788	0.467287	0.028*	
C15	0.6761 (3)	0.3581 (2)	0.6803 (2)	0.0204 (6)	
H15A	0.637561	0.374313	0.733311	0.031*	
H15B	0.753543	0.341244	0.692906	0.031*	
H15C	0.638843	0.304102	0.653203	0.031*	
C16	0.7459 (2)	0.5622 (2)	0.66320 (19)	0.0140 (5)	
C17	0.7559 (2)	0.5680 (2)	0.7543 (2)	0.0174 (6)	
H17	0.726354	0.523459	0.793791	0.021*	
C18	0.8173 (3)	0.6514 (2)	0.7758 (2)	0.0196 (6)	
H18	0.835968	0.671817	0.831577	0.023*	
C19	0.8455 (2)	0.6984 (2)	0.6992 (2)	0.0202 (6)	
H19	0.885821	0.756244	0.694769	0.024*	
C20	0.8032 (2)	0.6442 (2)	0.6298 (2)	0.0157 (6)	
C21	0.8143 (2)	0.6704 (2)	0.5375 (2)	0.0186 (6)	
H21A	0.854177	0.731777	0.532712	0.022*	
H21B	0.858954	0.621447	0.507897	0.022*	
C22	0.6358 (3)	0.7504 (2)	0.5361 (3)	0.0308 (8)	
H22A	0.626762	0.735500	0.596616	0.046*	
H22B	0.562731	0.751317	0.508457	0.046*	
H22C	0.671115	0.812870	0.530116	0.046*	
C23	0.7193 (4)	0.6974 (3)	0.4060 (2)	0.0333 (8)	
H23A	0.761764	0.756243	0.398401	0.050*	
H23B	0.646017	0.704129	0.379302	0.050*	
H23C	0.759445	0.644476	0.379535	0.050*	
C24	0.9906 (3)	0.4417 (2)	0.7387 (2)	0.0257 (7)	
H24	0.959441	0.392924	0.773004	0.031*	
C25	1.0489 (3)	0.5225 (2)	0.7685 (2)	0.0219 (7)	
H25	1.064181	0.537180	0.826365	0.026*	
C26	1.0806 (2)	0.5777 (2)	0.6972 (2)	0.0242 (6)	
H26	1.120399	0.635990	0.698549	0.029*	
C27	1.0418 (3)	0.5297 (3)	0.6227 (2)	0.0297 (8)	
H27	1.051335	0.550430	0.565617	0.036*	
C28	0.9869 (3)	0.4461 (3)	0.6491 (2)	0.0301 (8)	
H28	0.953179	0.400581	0.612719	0.036*	

### (Rp,Rp)-Bis{2-[(dimethylamino)methyl]ferrocenyl}dimethylsilane (2) Atomic displacement parameters (Å^2^)

**Table:**

	*U*^11^	*U*^22^	*U*^33^	*U*^12^	*U*^13^	*U*^23^
Fe1	0.00972 (19)	0.01196 (19)	0.0194 (2)	−0.00185 (16)	−0.00041 (18)	−0.00196 (16)
Fe2	0.00825 (19)	0.0162 (2)	0.0170 (2)	−0.00009 (16)	0.00075 (18)	−0.00221 (17)
Si1	0.0106 (3)	0.0123 (3)	0.0160 (4)	−0.0005 (3)	0.0005 (3)	−0.0018 (3)
N1	0.0189 (13)	0.0324 (15)	0.0235 (15)	−0.0047 (11)	0.0036 (11)	−0.0099 (11)
N2	0.0197 (13)	0.0177 (12)	0.0303 (16)	−0.0027 (10)	−0.0049 (11)	0.0044 (11)
C1	0.0188 (14)	0.0123 (13)	0.0307 (18)	−0.0016 (11)	−0.0040 (13)	−0.0030 (13)
C2	0.0243 (16)	0.0168 (14)	0.0278 (18)	−0.0088 (12)	−0.0001 (14)	0.0005 (12)
C3	0.0157 (15)	0.0211 (15)	0.042 (2)	−0.0091 (12)	−0.0012 (14)	−0.0060 (15)
C4	0.0232 (16)	0.0192 (14)	0.0327 (19)	−0.0065 (12)	−0.0121 (14)	−0.0044 (13)
C5	0.0258 (17)	0.0167 (13)	0.0239 (16)	−0.0026 (12)	0.0014 (13)	−0.0050 (12)
C6	0.0139 (13)	0.0126 (12)	0.0215 (16)	−0.0027 (10)	−0.0016 (11)	0.0014 (11)
C7	0.0167 (14)	0.0144 (13)	0.0288 (17)	0.0009 (11)	−0.0052 (12)	−0.0005 (11)
C8	0.0137 (14)	0.0158 (13)	0.0332 (18)	0.0007 (11)	0.0016 (12)	−0.0071 (13)
C9	0.0126 (14)	0.0138 (12)	0.0248 (16)	−0.0016 (10)	0.0015 (12)	−0.0067 (11)
C10	0.0131 (13)	0.0121 (12)	0.0200 (16)	−0.0022 (10)	0.0021 (11)	−0.0022 (11)
C11	0.0165 (14)	0.0264 (14)	0.0226 (16)	−0.0061 (13)	0.0039 (13)	−0.0058 (12)
C12	0.039 (2)	0.0319 (19)	0.037 (2)	−0.0047 (16)	0.0037 (17)	−0.0168 (16)
C13	0.031 (2)	0.055 (2)	0.0202 (18)	−0.0029 (17)	0.0012 (15)	−0.0054 (16)
C14	0.0174 (13)	0.0187 (14)	0.0203 (15)	0.0019 (11)	0.0025 (11)	−0.0044 (12)
C15	0.0212 (15)	0.0166 (13)	0.0236 (17)	−0.0005 (12)	−0.0007 (13)	0.0012 (12)
C16	0.0078 (12)	0.0164 (13)	0.0178 (14)	0.0004 (10)	−0.0002 (10)	−0.0018 (11)
C17	0.0097 (12)	0.0233 (14)	0.0191 (15)	−0.0003 (11)	0.0026 (11)	−0.0056 (12)
C18	0.0132 (14)	0.0248 (15)	0.0207 (16)	0.0006 (12)	0.0018 (12)	−0.0128 (12)
C19	0.0141 (14)	0.0156 (13)	0.0308 (18)	−0.0002 (10)	−0.0003 (13)	−0.0069 (13)
C20	0.0102 (13)	0.0140 (13)	0.0229 (16)	0.0008 (10)	−0.0011 (11)	−0.0014 (11)
C21	0.0142 (14)	0.0156 (13)	0.0260 (17)	−0.0019 (11)	−0.0003 (12)	0.0033 (12)
C22	0.0191 (15)	0.0251 (16)	0.048 (2)	0.0028 (12)	−0.0058 (16)	0.0012 (16)
C23	0.044 (2)	0.0260 (17)	0.030 (2)	−0.0062 (15)	−0.0121 (17)	0.0113 (15)
C24	0.0162 (14)	0.0198 (14)	0.041 (2)	0.0037 (12)	0.0011 (14)	0.0032 (14)
C25	0.0139 (13)	0.0281 (16)	0.0237 (17)	0.0052 (12)	−0.0046 (12)	0.0007 (13)
C26	0.0083 (12)	0.0294 (15)	0.0350 (18)	−0.0011 (12)	0.0007 (13)	0.0045 (14)
C27	0.0156 (15)	0.052 (2)	0.0215 (18)	0.0138 (15)	0.0042 (13)	0.0007 (16)
C28	0.0178 (15)	0.0334 (18)	0.039 (2)	0.0126 (14)	−0.0078 (14)	−0.0182 (16)

### (Rp,Rp)-Bis{2-[(dimethylamino)methyl]ferrocenyl}dimethylsilane (2) Geometric parameters (Å, º)

**Table:**

Fe1—C1	2.056 (3)	C8—H8	0.9500
Fe1—C2	2.041 (3)	C8—C9	1.437 (4)
Fe1—C3	2.041 (3)	C9—C10	1.442 (4)
Fe1—C4	2.049 (3)	C9—C11	1.501 (5)
Fe1—C5	2.060 (3)	C11—H11A	0.9900
Fe1—C6	2.054 (3)	C11—H11B	0.9900
Fe1—C7	2.046 (3)	C12—H12A	0.9800
Fe1—C8	2.039 (3)	C12—H12B	0.9800
Fe1—C9	2.040 (3)	C12—H12C	0.9800
Fe1—C10	2.076 (3)	C13—H13A	0.9800
Fe2—C16	2.068 (3)	C13—H13B	0.9800
Fe2—C17	2.051 (3)	C13—H13C	0.9800
Fe2—C18	2.051 (3)	C14—H14A	0.9800
Fe2—C19	2.053 (3)	C14—H14B	0.9800
Fe2—C20	2.046 (3)	C14—H14C	0.9800
Fe2—C24	2.055 (3)	C15—H15A	0.9800
Fe2—C25	2.057 (3)	C15—H15B	0.9800
Fe2—C26	2.046 (3)	C15—H15C	0.9800
Fe2—C27	2.043 (3)	C16—C17	1.440 (4)
Fe2—C28	2.050 (3)	C16—C20	1.442 (4)
Si1—C10	1.874 (3)	C17—H17	0.9500
Si1—C14	1.870 (3)	C17—C18	1.427 (4)
Si1—C15	1.869 (3)	C18—H18	0.9500
Si1—C16	1.873 (3)	C18—C19	1.414 (5)
N1—C11	1.462 (4)	C19—H19	0.9500
N1—C12	1.455 (5)	C19—C20	1.424 (4)
N1—C13	1.446 (5)	C20—C21	1.502 (5)
N2—C21	1.464 (4)	C21—H21A	0.9900
N2—C22	1.453 (5)	C21—H21B	0.9900
N2—C23	1.452 (5)	C22—H22A	0.9800
C1—H1	0.9500	C22—H22B	0.9800
C1—C2	1.427 (5)	C22—H22C	0.9800
C1—C5	1.420 (5)	C23—H23A	0.9800
C2—H2	0.9500	C23—H23B	0.9800
C2—C3	1.418 (5)	C23—H23C	0.9800
C3—H3	0.9500	C24—H24	0.9500
C3—C4	1.423 (6)	C24—C25	1.415 (5)
C4—H4	0.9500	C24—C28	1.410 (5)
C4—C5	1.421 (5)	C25—H25	0.9500
C5—H5	0.9500	C25—C26	1.416 (5)
C6—H6	0.9500	C26—H26	0.9500
C6—C7	1.422 (4)	C26—C27	1.429 (5)
C6—C10	1.434 (4)	C27—H27	0.9500
C7—H7	0.9500	C27—C28	1.411 (6)
C7—C8	1.423 (5)	C28—H28	0.9500
			
C1—Fe1—C5	40.36 (14)	C7—C6—C10	109.5 (3)
C1—Fe1—C10	109.75 (12)	C10—C6—Fe1	70.48 (16)
C2—Fe1—C1	40.77 (14)	C10—C6—H6	125.2
C2—Fe1—C3	40.66 (14)	Fe1—C7—H7	126.0
C2—Fe1—C4	68.32 (14)	C6—C7—Fe1	70.02 (16)
C2—Fe1—C5	68.20 (14)	C6—C7—H7	126.2
C2—Fe1—C6	161.37 (13)	C6—C7—C8	107.7 (3)
C2—Fe1—C7	155.59 (13)	C8—C7—Fe1	69.36 (17)
C2—Fe1—C10	123.40 (13)	C8—C7—H7	126.2
C3—Fe1—C1	68.47 (13)	Fe1—C8—H8	126.4
C3—Fe1—C4	40.73 (16)	C7—C8—Fe1	69.87 (17)
C3—Fe1—C5	68.31 (14)	C7—C8—H8	125.9
C3—Fe1—C6	157.77 (13)	C7—C8—C9	108.2 (3)
C3—Fe1—C7	119.98 (13)	C9—C8—Fe1	69.41 (17)
C3—Fe1—C10	157.81 (14)	C9—C8—H8	125.9
C4—Fe1—C1	68.15 (13)	C8—C9—Fe1	69.35 (17)
C4—Fe1—C5	40.45 (14)	C8—C9—C10	108.3 (3)
C4—Fe1—C6	124.23 (13)	C8—C9—C11	124.6 (3)
C4—Fe1—C10	160.90 (14)	C10—C9—Fe1	70.82 (17)
C5—Fe1—C10	125.64 (13)	C10—C9—C11	127.1 (3)
C6—Fe1—C1	126.61 (13)	C11—C9—Fe1	125.1 (2)
C6—Fe1—C5	111.23 (13)	Si1—C10—Fe1	128.94 (15)
C6—Fe1—C10	40.65 (12)	C6—C10—Fe1	68.87 (16)
C7—Fe1—C1	161.64 (14)	C6—C10—Si1	122.8 (2)
C7—Fe1—C4	106.75 (13)	C6—C10—C9	106.3 (3)
C7—Fe1—C5	124.49 (13)	C9—C10—Fe1	68.18 (16)
C7—Fe1—C6	40.58 (12)	C9—C10—Si1	130.8 (2)
C7—Fe1—C10	68.95 (11)	N1—C11—C9	112.0 (3)
C8—Fe1—C1	157.25 (14)	N1—C11—H11A	109.2
C8—Fe1—C2	119.58 (14)	N1—C11—H11B	109.2
C8—Fe1—C3	103.68 (13)	C9—C11—H11A	109.2
C8—Fe1—C4	120.60 (14)	C9—C11—H11B	109.2
C8—Fe1—C5	158.37 (14)	H11A—C11—H11B	107.9
C8—Fe1—C6	68.25 (12)	N1—C12—H12A	109.5
C8—Fe1—C7	40.76 (13)	N1—C12—H12B	109.5
C8—Fe1—C9	41.25 (13)	N1—C12—H12C	109.5
C8—Fe1—C10	69.08 (12)	H12A—C12—H12B	109.5
C9—Fe1—C1	122.80 (13)	H12A—C12—H12C	109.5
C9—Fe1—C2	105.50 (13)	H12B—C12—H12C	109.5
C9—Fe1—C3	119.96 (14)	N1—C13—H13A	109.5
C9—Fe1—C4	156.58 (13)	N1—C13—H13B	109.5
C9—Fe1—C5	160.20 (13)	N1—C13—H13C	109.5
C9—Fe1—C6	68.43 (12)	H13A—C13—H13B	109.5
C9—Fe1—C7	69.06 (12)	H13A—C13—H13C	109.5
C9—Fe1—C10	41.00 (12)	H13B—C13—H13C	109.5
C17—Fe2—C16	40.91 (12)	Si1—C14—H14A	109.5
C17—Fe2—C18	40.70 (12)	Si1—C14—H14B	109.5
C17—Fe2—C19	68.06 (13)	Si1—C14—H14C	109.5
C17—Fe2—C24	108.96 (13)	H14A—C14—H14B	109.5
C17—Fe2—C25	121.53 (13)	H14A—C14—H14C	109.5
C18—Fe2—C16	68.97 (12)	H14B—C14—H14C	109.5
C18—Fe2—C19	40.30 (14)	Si1—C15—H15A	109.5
C18—Fe2—C24	125.95 (15)	Si1—C15—H15B	109.5
C18—Fe2—C25	107.97 (13)	Si1—C15—H15C	109.5
C19—Fe2—C16	68.80 (12)	H15A—C15—H15B	109.5
C19—Fe2—C24	162.07 (14)	H15A—C15—H15C	109.5
C19—Fe2—C25	124.93 (13)	H15B—C15—H15C	109.5
C20—Fe2—C16	41.04 (11)	Si1—C16—Fe2	125.98 (15)
C20—Fe2—C17	68.40 (12)	C17—C16—Fe2	68.94 (16)
C20—Fe2—C18	68.38 (13)	C17—C16—Si1	123.4 (2)
C20—Fe2—C19	40.66 (12)	C17—C16—C20	106.1 (3)
C20—Fe2—C24	156.37 (13)	C20—C16—Fe2	68.69 (15)
C20—Fe2—C25	161.45 (13)	C20—C16—Si1	130.4 (2)
C20—Fe2—C28	121.11 (14)	Fe2—C17—H17	126.2
C24—Fe2—C16	121.38 (13)	C16—C17—Fe2	70.15 (16)
C24—Fe2—C25	40.27 (14)	C16—C17—H17	125.5
C25—Fe2—C16	156.39 (13)	C18—C17—Fe2	69.65 (17)
C26—Fe2—C16	161.74 (13)	C18—C17—C16	108.9 (3)
C26—Fe2—C17	155.67 (13)	C18—C17—H17	125.5
C26—Fe2—C18	120.12 (13)	Fe2—C18—H18	126.0
C26—Fe2—C19	107.04 (13)	C17—C18—Fe2	69.65 (16)
C26—Fe2—C20	124.39 (13)	C17—C18—H18	126.0
C26—Fe2—C24	67.96 (13)	C19—C18—Fe2	69.90 (17)
C26—Fe2—C25	40.37 (13)	C19—C18—C17	107.9 (3)
C26—Fe2—C28	68.10 (14)	C19—C18—H18	126.0
C27—Fe2—C16	124.63 (14)	Fe2—C19—H19	126.6
C27—Fe2—C17	162.38 (14)	C18—C19—Fe2	69.79 (18)
C27—Fe2—C18	155.23 (15)	C18—C19—H19	125.8
C27—Fe2—C19	120.43 (15)	C18—C19—C20	108.4 (3)
C27—Fe2—C20	107.13 (14)	C20—C19—Fe2	69.42 (16)
C27—Fe2—C24	67.88 (15)	C20—C19—H19	125.8
C27—Fe2—C25	68.11 (14)	C16—C20—Fe2	70.27 (16)
C27—Fe2—C26	40.91 (15)	C16—C20—C21	126.2 (3)
C27—Fe2—C28	40.32 (16)	C19—C20—Fe2	69.92 (17)
C28—Fe2—C16	107.98 (13)	C19—C20—C16	108.6 (3)
C28—Fe2—C17	126.17 (14)	C19—C20—C21	125.2 (3)
C28—Fe2—C18	162.91 (15)	C21—C20—Fe2	127.0 (2)
C28—Fe2—C19	155.80 (16)	N2—C21—C20	111.6 (3)
C28—Fe2—C24	40.16 (15)	N2—C21—H21A	109.3
C28—Fe2—C25	67.68 (13)	N2—C21—H21B	109.3
C14—Si1—C10	105.43 (14)	C20—C21—H21A	109.3
C14—Si1—C16	112.17 (13)	C20—C21—H21B	109.3
C15—Si1—C10	111.48 (14)	H21A—C21—H21B	108.0
C15—Si1—C14	109.98 (15)	N2—C22—H22A	109.5
C15—Si1—C16	106.74 (14)	N2—C22—H22B	109.5
C16—Si1—C10	111.12 (12)	N2—C22—H22C	109.5
C12—N1—C11	111.4 (3)	H22A—C22—H22B	109.5
C13—N1—C11	110.3 (3)	H22A—C22—H22C	109.5
C13—N1—C12	111.0 (3)	H22B—C22—H22C	109.5
C22—N2—C21	112.2 (3)	N2—C23—H23A	109.5
C23—N2—C21	110.1 (3)	N2—C23—H23B	109.5
C23—N2—C22	111.1 (3)	N2—C23—H23C	109.5
Fe1—C1—H1	126.4	H23A—C23—H23B	109.5
C2—C1—Fe1	69.02 (17)	H23A—C23—H23C	109.5
C2—C1—H1	126.1	H23B—C23—H23C	109.5
C5—C1—Fe1	69.97 (17)	Fe2—C24—H24	125.9
C5—C1—H1	126.1	C25—C24—Fe2	69.95 (19)
C5—C1—C2	107.7 (3)	C25—C24—H24	125.9
Fe1—C2—H2	125.8	C28—C24—Fe2	69.7 (2)
C1—C2—Fe1	70.21 (17)	C28—C24—H24	125.9
C1—C2—H2	125.9	C28—C24—C25	108.1 (3)
C3—C2—Fe1	69.69 (18)	Fe2—C25—H25	126.4
C3—C2—C1	108.2 (3)	C24—C25—Fe2	69.78 (18)
C3—C2—H2	125.9	C24—C25—H25	126.0
Fe1—C3—H3	125.9	C24—C25—C26	108.1 (3)
C2—C3—Fe1	69.64 (18)	C26—C25—Fe2	69.38 (17)
C2—C3—H3	126.1	C26—C25—H25	126.0
C2—C3—C4	107.8 (3)	Fe2—C26—H26	125.7
C4—C3—Fe1	69.93 (18)	C25—C26—Fe2	70.24 (17)
C4—C3—H3	126.1	C25—C26—H26	126.2
Fe1—C4—H4	126.1	C25—C26—C27	107.6 (3)
C3—C4—Fe1	69.35 (18)	C27—C26—Fe2	69.44 (18)
C3—C4—H4	125.9	C27—C26—H26	126.2
C5—C4—Fe1	70.20 (18)	Fe2—C27—H27	125.7
C5—C4—C3	108.1 (3)	C26—C27—Fe2	69.65 (18)
C5—C4—H4	125.9	C26—C27—H27	126.1
Fe1—C5—H5	126.6	C28—C27—Fe2	70.12 (19)
C1—C5—Fe1	69.67 (17)	C28—C27—C26	107.7 (3)
C1—C5—C4	108.1 (3)	C28—C27—H27	126.1
C1—C5—H5	125.9	Fe2—C28—H28	126.1
C4—C5—Fe1	69.34 (18)	C24—C28—Fe2	70.1 (2)
C4—C5—H5	125.9	C24—C28—C27	108.4 (3)
Fe1—C6—H6	126.5	C24—C28—H28	125.8
C7—C6—Fe1	69.40 (17)	C27—C28—Fe2	69.6 (2)
C7—C6—H6	125.2	C27—C28—H28	125.8
			
Fe1—C1—C2—C3	59.5 (2)	C8—C9—C11—N1	−121.0 (3)
Fe1—C1—C5—C4	−58.9 (2)	C10—Si1—C16—Fe2	−175.33 (17)
Fe1—C2—C3—C4	59.7 (2)	C10—Si1—C16—C17	97.7 (3)
Fe1—C3—C4—C5	59.7 (2)	C10—Si1—C16—C20	−83.7 (3)
Fe1—C4—C5—C1	59.1 (2)	C10—C6—C7—Fe1	−59.2 (2)
Fe1—C6—C7—C8	59.4 (2)	C10—C6—C7—C8	0.2 (3)
Fe1—C6—C10—Si1	123.5 (2)	C10—C9—C11—N1	59.6 (4)
Fe1—C6—C10—C9	−58.09 (19)	C11—C9—C10—Fe1	120.0 (3)
Fe1—C7—C8—C9	59.0 (2)	C11—C9—C10—Si1	−3.3 (4)
Fe1—C8—C9—C10	60.4 (2)	C11—C9—C10—C6	178.5 (3)
Fe1—C8—C9—C11	−119.1 (3)	C12—N1—C11—C9	57.9 (4)
Fe1—C9—C10—Si1	−123.3 (2)	C13—N1—C11—C9	−178.4 (3)
Fe1—C9—C10—C6	58.53 (19)	C14—Si1—C10—Fe1	64.7 (2)
Fe1—C9—C11—N1	151.2 (2)	C14—Si1—C10—C6	−23.6 (3)
Fe2—C16—C17—C18	59.0 (2)	C14—Si1—C10—C9	158.4 (3)
Fe2—C16—C20—C19	−59.6 (2)	C14—Si1—C16—Fe2	−57.6 (2)
Fe2—C16—C20—C21	122.0 (3)	C14—Si1—C16—C17	−144.6 (2)
Fe2—C17—C18—C19	59.7 (2)	C14—Si1—C16—C20	34.1 (3)
Fe2—C18—C19—C20	58.8 (2)	C15—Si1—C10—Fe1	−54.7 (2)
Fe2—C19—C20—C16	59.8 (2)	C15—Si1—C10—C6	−142.9 (2)
Fe2—C19—C20—C21	−121.7 (3)	C15—Si1—C10—C9	39.1 (3)
Fe2—C20—C21—N2	147.6 (2)	C15—Si1—C16—Fe2	62.9 (2)
Fe2—C24—C25—C26	−59.0 (2)	C15—Si1—C16—C17	−24.0 (3)
Fe2—C24—C28—C27	59.2 (2)	C15—Si1—C16—C20	154.6 (3)
Fe2—C25—C26—C27	−59.6 (2)	C16—Si1—C10—Fe1	−173.59 (18)
Fe2—C26—C27—C28	−60.0 (2)	C16—Si1—C10—C6	98.1 (3)
Fe2—C27—C28—C24	−59.5 (2)	C16—Si1—C10—C9	−79.8 (3)
Si1—C16—C17—Fe2	120.0 (2)	C16—C17—C18—Fe2	−59.3 (2)
Si1—C16—C17—C18	179.1 (2)	C16—C17—C18—C19	0.3 (3)
Si1—C16—C20—Fe2	−119.7 (2)	C16—C20—C21—N2	56.0 (4)
Si1—C16—C20—C19	−179.4 (2)	C17—C16—C20—Fe2	59.05 (19)
Si1—C16—C20—C21	2.2 (4)	C17—C16—C20—C19	−0.6 (3)
C1—C2—C3—Fe1	−59.9 (2)	C17—C16—C20—C21	−179.0 (3)
C1—C2—C3—C4	−0.1 (3)	C17—C18—C19—Fe2	−59.5 (2)
C2—C1—C5—Fe1	58.9 (2)	C17—C18—C19—C20	−0.7 (3)
C2—C1—C5—C4	0.0 (3)	C18—C19—C20—Fe2	−59.1 (2)
C2—C3—C4—Fe1	−59.6 (2)	C18—C19—C20—C16	0.8 (3)
C2—C3—C4—C5	0.2 (3)	C18—C19—C20—C21	179.2 (3)
C3—C4—C5—Fe1	−59.2 (2)	C19—C20—C21—N2	−122.1 (3)
C3—C4—C5—C1	−0.1 (3)	C20—C16—C17—Fe2	−58.89 (19)
C5—C1—C2—Fe1	−59.5 (2)	C20—C16—C17—C18	0.2 (3)
C5—C1—C2—C3	0.1 (3)	C22—N2—C21—C20	59.7 (3)
C6—C7—C8—Fe1	−59.8 (2)	C23—N2—C21—C20	−176.0 (3)
C6—C7—C8—C9	−0.8 (3)	C24—C25—C26—Fe2	59.3 (2)
C7—C6—C10—Fe1	58.5 (2)	C24—C25—C26—C27	−0.4 (3)
C7—C6—C10—Si1	−177.9 (2)	C25—C24—C28—Fe2	−59.6 (2)
C7—C6—C10—C9	0.5 (3)	C25—C24—C28—C27	−0.5 (4)
C7—C8—C9—Fe1	−59.3 (2)	C25—C26—C27—Fe2	60.1 (2)
C7—C8—C9—C10	1.1 (3)	C25—C26—C27—C28	0.1 (4)
C7—C8—C9—C11	−178.4 (3)	C26—C27—C28—Fe2	59.7 (2)
C8—C9—C10—Fe1	−59.5 (2)	C26—C27—C28—C24	0.2 (4)
C8—C9—C10—Si1	177.3 (2)	C28—C24—C25—Fe2	59.5 (2)
C8—C9—C10—C6	−0.9 (3)	C28—C24—C25—C26	0.5 (4)
